# Antibiofilm and Antimicrobial-Enhancing Activity of *Chelidonium majus* and *Corydalis cheilanthifolia* Extracts against Multidrug-Resistant *Helicobacter pylori*

**DOI:** 10.3390/pathogens10081033

**Published:** 2021-08-16

**Authors:** Paweł Krzyżek, Adam Junka, Wojciech Słupski, Arleta Dołowacka-Jóźwiak, Bartosz J. Płachno, Aleksandra Sobiecka, Adam Matkowski, Grzegorz Chodaczek, Tadeusz Płusa, Grażyna Gościniak, Sylwia Zielińska

**Affiliations:** 1 Department of Medical Microbiology, Wroclaw Medical University, 50-368 Wroclaw, Poland; grazyna.gosciniak@umed.wroc.pl; 2 Department of Pharmaceutical Microbiology and Parasitology, Wroclaw Medical University, 50-556 Wroclaw, Poland; adam.junka@umed.wroc.pl; 3 Department of Pharmacology, Wroclaw Medical University, 50-345 Wrocław, Poland; wojciech.slupski@umed.wroc.pl; 4 Department of Drug Form Technology, Wroclaw Medical University, 50-556 Wroclaw, Poland; arleta.dolowacka@umed.wroc.pl; 5 Department of Plant Cytology and Embryology, Institute of Botany, Faculty of Biology, Jagiellonian University in Cracow, 9 Gronostajowa St., 30-387 Cracow, Poland; bartosz.plachno@uj.edu.pl; 6 Department of Pharmaceutical Biotechnology, Wroclaw Medical University, 50-556 Wrocław, Poland; aleksandra.sobiecka@umed.wroc.pl (A.S.); sylwia.zielinska@umed.wroc.pl (S.Z.); 7 Department of Pharmaceutical Biology and Botanical Garden of Medicinal Plants, Wroclaw Medical University, 50-556 Wroclaw, Poland; adam.matkowski@umed.wroc.pl; 8 Bioimaging Laboratory, Łukasiewicz Research Network—PORT Polish Center for Technology Development, 54-066 Wroclaw, Poland; grzegorz.chodaczek@port.org.pl; 9 Faculty of Medicine, Lazarski University, 02-662 Warszawa, Poland; tadeusz.plusa@lazarski.pl

**Keywords:** *Helicobacter pylori*, biofilm, synergism, Bioflux, flow system, Papaveraceae, amoxicillin, 3-bromopyruvate, sertraline, bacterial cellulose

## Abstract

*Helicobacter pylori* is a Gram-negative bacterium that colonizes the stomach of about 60% of people worldwide. The search for new drugs with activity against *H. pylori* is now a hotspot in the effective and safe control of this bacterium. Therefore, the aim of this research was to determine the antibacterial activity of extracts from selected plants of the Papaveraceae family against planktonic and biofilm forms of the multidrug-resistant clinical strain of *H. pylori* using a broad spectrum of analytical in vitro methods. It was revealed that among the tested extracts, those obtained from *Corydalis cheilanthifolia* and *Chelidonium majus* were the most active, with minimal inhibitory concentrations (MICs) of 64 µg/mL and 128 µg/mL, respectively. High concentrations of both extracts showed cytotoxicity against cell lines of human hepatic origin. Therefore, we attempted to lower their MICs through the use of a synergistic combination with synthetic antimicrobials as well as by applying cellulose as a drug carrier. Using checkerboard assays, we determined that both extracts presented synergistic interactions with amoxicillin (AMX) and 3-bromopyruvate (3-BP) (FICI = 0.5) and additive relationships with sertraline (SER) (FICI = 0.75). The antibiofilm activity of extracts and their combinations with AMX, 3-BP, or SER, was analyzed by two methods, i.e., the microcapillary overgrowth under flow conditions (the Bioflux system) and assessment of the viability of lawn biofilms after exposure to drugs released from bacterial cellulose (BC) carriers. Using both methods, we observed a several-fold decrease in the level of *H. pylori* biofilm, indicating the ability of the tested compounds to eradicate the microbial biofilm. The obtained results indicate that application of plant-derived extracts from the Papaveraceae family combined with synthetic antimicrobials, absorbed into organic BC carrier, may be considered a promising way of fighting biofilm-forming *H. pylori*.

## 1. Introduction

*Helicobacter pylori* (*H. pylori*) is a Gram-negative microaerophilic bacterium that colonizes the stomach of about 60% of people worldwide [1,2]. In the absence of appropriate therapy, this microorganism is able to persist life-long in the host. The development of diseases related to the presence of the aforementioned pathogen, including gastric ulcers or neoplasms, is of multifactorial character, and it depends on the strain’s virulence, genetic predisposition, the efficiency of the host’s immune system, and environmental factors (sanitation, diet, addictions) [1,3]. Taking into account the prevalence and mortality caused by *H. pylori* infections, it seems that appropriate therapy is crucial to prevent patients from developing future health complications [3].

Soon after the discovery of *H. pylori* in the 1980s, a high sensitivity of this bacterium to most of the classically used antibiotics was revealed [4]. However, this favorable phenomenon was rapidly changed due to observed worldwide, growing antibiotic resistance. Currently, the treatment of *H. pylori* has reached the point where the majority of traditional methods of its eradication have lost their effectiveness [3]. *H. pylori* antibiotic resistance is the main cause of these therapeutic failures and a major challenge for clinicians [5]. The latest data on *H. pylori* antibiotic resistance in Europe showed that the level of primary resistance of this bacterium was 21.8% for clarithromycin (CLR), 15.8% for levofloxacin (LEV), and 38.9% for metronidazole (MTZ) [5]. The results of this European study are consistent with both world-level [6] and national-level data published by our team [7]. Therefore, attention should be paid to the maintenance of an appropriate degree of *H. pylori* eradication and development of alternative, effective methods for combating this pathogen [8,9,10].

The search for new drugs displaying activity against *H. pylori* is now a hotspot in the effective and safe control of this bacterium [11]. High hopes are associated with phytotherapy, which is the practice of using raw or processed plant products (flowers, leaves, stalks, roots, or seeds) for medicinal purposes [12,13]. The vast majority of plant medicine has an empirical basis, resulting from the multigenerational use of specific plants in relieving the symptoms of given diseases [12]. Plant extracts that are used classically in the treatment of gastrointestinal ailments can be thus scrutinized to find drugs that are potent against *H. pylori* [13,14]. These extracts contain a broad range of different phytochemicals (alkaloids, flavonoids, saponins, and terpenes), usually being specialized plant metabolites with important physiological functions [15]. For this reason, the isolation and identification of bioactive plant compounds is currently one of the most important areas aimed at finding new drugs for combating *H. pylori* infections [13].

Over the past decades, pharmacological and biochemical studies of metabolites from the Papaveraceae family have shed light on their potential use in many sectors of medicine. Papaveraceae, the poppy family of flowering plants, are of great interest in the context of their medical use, which is related to their ability to produce numerous alkaloids with significant bioactivity [16]. The biological properties of these compounds include antitumor, antimicrobial, and anti-inflammatory activity [16,17]. All of these features may turn out to be particularly beneficial in the context of combating antibiotic-resistant strains of *H. pylori* and eliminating the negative effects resulting from stomach colonization (the induction of gastric inflammation, ulcerations, and carcinogenesis). Therefore, the aim of this research was to determine the antibacterial activity of extracts from selected plants of the Papaveraceae family against planktonic and biofilm forms of the multidrug-resistant clinical strain of *H. pylori* and assessment of combined activity of these extracts with standardly applied and novel molecules of proven anti-*H. pylori* mode of action.

## 2. Results and Discussion

Based on a review of the literature and our previous results showing the antimicrobial activity of plant extracts from the Papaveraceae family [18,19,20,21], in the present research article we decided to verify this activity against *H. pylori* because such analyses were not performed in this respect so far. For this purpose, we chose the multidrug-resistant *H. pylori* 8064 strain, characterized by resistance to all three classically used antibiotics (CLR, LEV, and MTZ) but sensitive to amoxicillin (AMX) [22].

### 2.1. Antibacterial Activity against Planktonic Forms

In the first stage of our research, we decided to check the antibacterial activity of selected extracts from aerial and underground parts of Papaveraceae plants against *H. pylori* by determining minimal inhibitory concentration (MIC) and minimal bactericidal concentration (MBC) values (Table 1). It turned out that most of the tested extracts showed no antimicrobial activity against the *H. pylori* 8064 strain (both MICs and MBCs being ≥512 µg/mL). The only ones that showed any antibacterial action were aerial (A5/1; both MIC and MBC were 64 µg/mL) and underground parts (A5/2; MIC and MBC equal to 64 µg/mL and 128 µg/mL, respectively) of *Corydalis cheilanthifolia* and the underground parts (A4/2; MIC and MBC were 128 µg/mL and 256 µg/mL, respectively) of *Chelidonium majus*.

To confirm their antibacterial effect, the extracts were spotted on paper disks placed on the inoculum of *H. pylori* spread over agar plate. After an incubation time, the zones of microbial growth inhibition around paper disks were measured (Table 2 and Figure S1). These were equal to 22.7 ± 2.5 mm for A4/2 and 30.7 ± 0.6 mm and 28 ± 1 mm for A5/1 and A5/2, respectively. In comparison, paper disks with CLR, LEV, and MTZ, antibiotics to which *H. pylori* 8064 is resistant, had no or had marginal effect on the growth (11.3 ± 1.5 mm, 6 mm, and 6 mm, respectively). The application of disks with AMX, an antibiotic with high activity against this strain, contributed to the appearance of a large growth inhibition zone (55.7 ± 2.1 mm). As many experts point out, the frequency of overuse and inappropriate prescription of antibiotics in the treatment of bacterial infections throws a shadow on the possible future utilization of many of them [4,8,9,23]. This issue is of particular importance because over the last 20 years a rapid increase in antibiotic resistance of *H. pylori* has been observed, and a very limited array of antibiotics are left in the treatment of multidrug-resistant strains of this bacterium [5,23]. Therefore, intensive search for new compounds displaying anti-*H. pylori* activity (as performed in this study) is of pivotal usability with regard to future treatment algorithms.

### 2.2. Cytotoxic Activity against Stomach- and Liver-Derived Cell Lines

Encouraged by the screening results, we chose the two extracts displaying the highest antimicrobial activity—A4/2 and A5/1 (from *C. majus* roots and *C. cheilanthifolia* herb, respectively) and decided to check their cytotoxic effect against stomach and liver cells. We noticed that the higher concentrations of both extracts had a cytotoxic effect on both cell lines, while their destructive effect was significantly lower for gastric cells (50% lethal concentration (LC_50_) was > 12,500 µg/mL for both) than for hepatic cells (LC_50_ was equal to ≥391 µg/mL for A5/1 and ≥98 µg/mL for A4/2) (Figure S2). We reviewed the literature on this aspect and we did not find any reports of gastric toxicity of these plant extracts. On the contrary, even a gastroprotective effect was mentioned [24,25], but we cannot say the same for liver cells. Several cases of acute hepatitis after ingestion of high doses of preparations containing *C. majus* and two *Corydalis* species (*C. yanhusuo, C. remota*) have been reported [26,27,28]. Since these were rather rare cases, we attempted to reduce their MIC/MBC values and potential toxicity through the use of synergistic treatment [29,30] and drug carriers [31,32], two methods known for reducing drug side effects.

### 2.3. Synergistic Activity of Extracts and Synthetic Drugs

**Figure 1:**
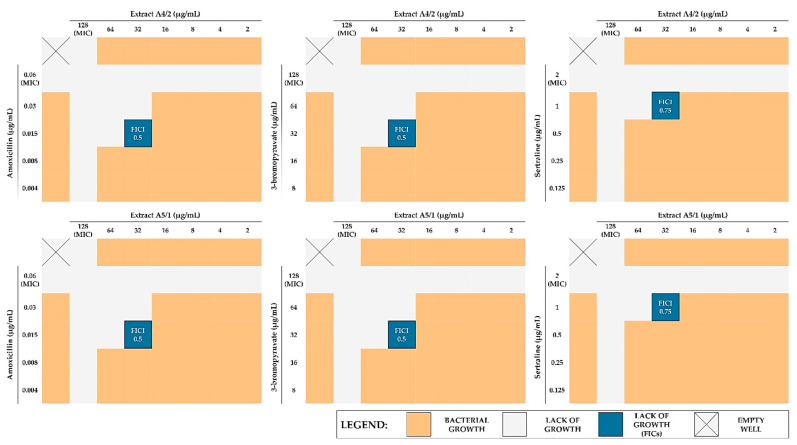
Graphical presentation of checkerboard assays between selected plant extracts (A4/2 and A5/1) and selected synthetic substances (amoxicillin, 3-bromopyruvate, and sertraline) against *H. pylori* 8064. Abbreviations: MIC, minimal inhibitory concentration; FICI, fractional inhibitory concentration index.

Synergistic approaches aimed at treating infections caused by *H. pylori* are currently of high hopes (a literature review by Krzyżek et al. (2020) [33]). Using a checkerboard assay, we determined the existence of interactions between extracts (A4/2 and A5/1) and synthetic substances with proven antibacterial activity against *H. pylori*, i.e., the routinely used antibiotic AMX, as well as sertraline (SER) and 3-bromopyruvate (3-BP), which we have recently studied for their anti-*H. pylori* properties [22,34,35]. Interestingly, an identical type of interaction for both extracts coupled with the abovementioned synthetic antimicrobials was observed (Table 3 and Figure 1). In the case of AMX and 3-BP, the interaction with the extracts was synergistic (FICI = 0.5) and allowed us to reduce by 4-fold the MIC values of both components. The interaction of the extracts with SER was additive (FICI = 0.75), although in this case it was also possible to reduce the MIC of the extracts by 4 times. Since an identical type of interaction for the extracts of both plants (*C. cheilanthifolia* and *C. majus*) was detected, we suspected that this phenomenon may rely on their similar antimicrobial mechanism of action.

The phytochemical analyses previously conducted for the studied plants showed that they contain different proportions of various phenolic compounds, but most of all they contain isoquinoline alkaloids, such as protoberberine, protopine, and benzophenanthridine derivatives [18,19,36,37,38]. This is a large class of alkaloids that occurs in several plant families, such as Papaveraceae, Fumariaceae, Berberidaceae, and Rutaceae [39]. Most often, berberine, coptisine, protopine, and sanguinarine predominate, but their content in individual plant organs is variable. The abundance of these compounds can be influenced by different factors such as harvest time, edaphic factors, or a challenge by pathogens and herbivores [32]. The direction of biological activity depends not only on the amount of plant compounds contained in the extracts but above all on their structure and co-existence with other, often different groups of substances [26]. The mechanism of antimicrobial activity of alkaloids is multi-directional, but the most important in the context of synergistic therapy is the ability to disrupt membrane integrity [40,41]. It seems that this mechanism may also be important in the case of positive interactions with the synthetic compounds we investigated. AMX and 3-BP are substances that enter microbial cells via membrane transport proteins [42,43], so that disruption of the integrity of cell membrane(s) may open additional portals for both of these compounds to enter the bacteria. Due to its lipophilicity, SER shows the ability to cross the membrane barrier independent of porins [44], and hence perhaps, the presence of isoquinoline alkaloids from both tested extracts may be less significant in enhancing its antimicrobial activity.

### 2.4. Anti-Biofilm Activity

Microbial biofilm is a multicellular structure composed of microbial cells embedded within biomatrix that may consist, in various proportions, of polysaccharides, proteins, lipids, and eDNA [45,46]. Scientists around the world highlight a crucial role of biofilms in therapeutic failures and the ability of biofilm-forming microorganisms to persistently colonize both inanimate objects and host tissues [47,48]. The amount of research concerning the production and meaning of *H. pylori* biofilm in pathogenesis of infections has also increased significantly in recent years (a literature review by Krzyżek et al. (2020) [49]). Noteworthy, it has been observed that the strain applied in our study, *H. pylori* 8064, is able to form strong biofilm structure in vitro [49].

**Figure 2:**
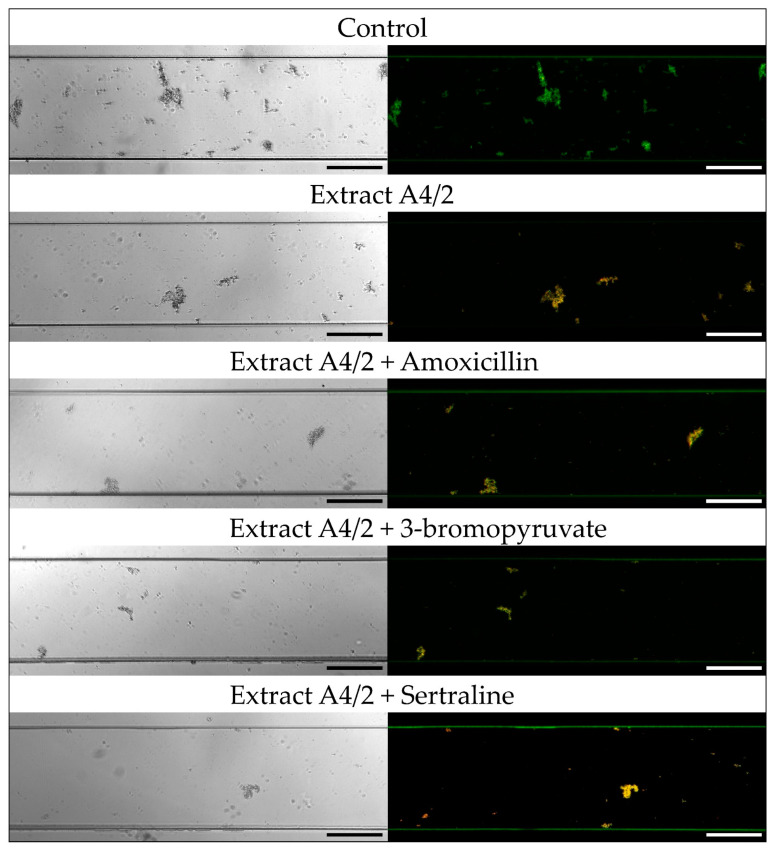
Representative light and fluorescence microscopy pictures taken at ×10 magnification of *H. pylori* 8064 strain treated with plant extract A4/2 and its combination with tested synthetic compounds (amoxicillin (AMX), 3-bromopyruvate (3-BP), or sertraline (SER)). Bacteria were treated with substances as follows: MIC of a plant extract, ¼×MIC of a plant extract + ¼ × MIC of AMX, ¼×MIC of a plant extract + ¼ × MIC of 3-BP, or ¼×MIC of a plant extract + ½×MIC of SER. The study controls were microcapillaries colonized by bacteria not exposed to any antimicrobial substances. In the case of fluorescence pictures, bacteria were stained with the LIVE/DEAD kit, in which green and yellow/orange fluorescence indicates live and damaged/dead bacteria, respectively. Scale bars show 20 µm.

**Figure 3:**
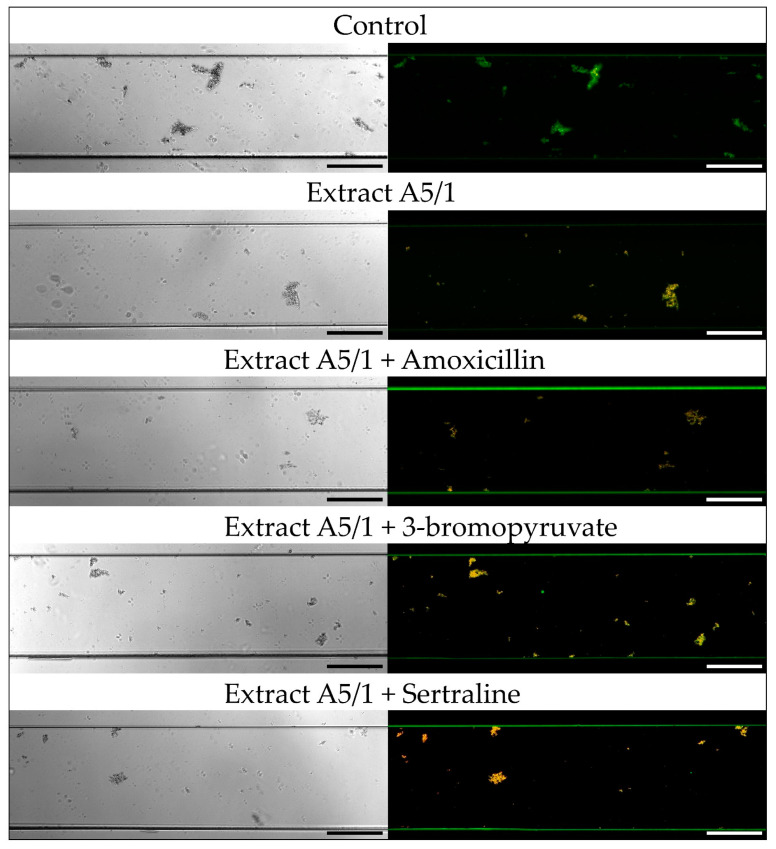
Representative light and fluorescence microscopy pictures taken at ×10 magnification of *H. pylori* 8064 strain treated with plant extract A5/1 and its combination with tested synthetic compounds (amoxicillin (AMX), 3-bromopyruvate (3-BP), or sertraline (SER)). Bacteria were treated with substances as follows: MIC of a plant extract, ¼ × MIC of a plant extract + ¼ × MIC of AMX, ¼ × MIC of a plant extract + ¼ × MIC of 3-BP, or ¼ × MIC of a plant extract + ½ × MIC of SER. The study controls were microcapillaries colonized by bacteria not exposed to any antimicrobial substances. In the case of fluorescence pictures, bacteria were stained with the LIVE/DEAD kit, in which green and yellow/orange fluorescence indicates live and damaged/dead bacteria, respectively. Scale bars show 20 µm.

**Figure 4:**
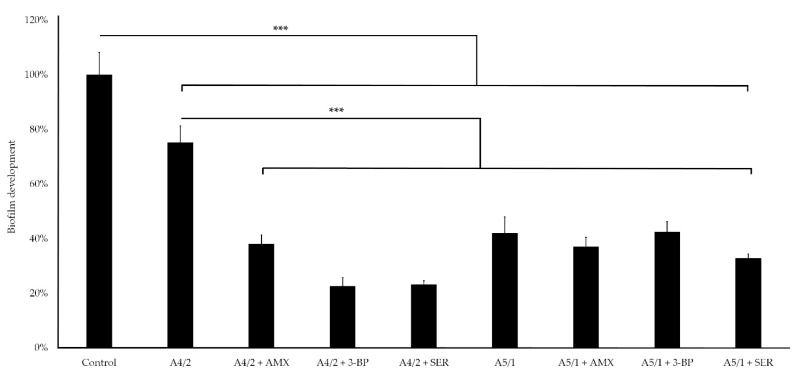
Degree of biofilm development of *H. pylori* 8064 treated with the tested plant extracts (A4/2 or A5/1) and their combination with tested synthetic compounds (amoxicillin (AMX), 3-bromopyruvate (3-BP), or sertraline (SER)) during the Bioflux-generated flow conditions. Bacteria were treated with substances as follows: MIC of a plant extract, ¼ × MIC of a plant extract + ¼ × MIC of AMX, ¼ × MIC of a plant extract + ¼ × MIC of 3-BP, or ¼ × MIC of a plant extract + ½ × MIC of SER. The study controls were microcapillaries colonized by bacteria not exposed to any antimicrobial substances. The *p*-value represented by *** is equal to <0.0001.

**Figure 5:**
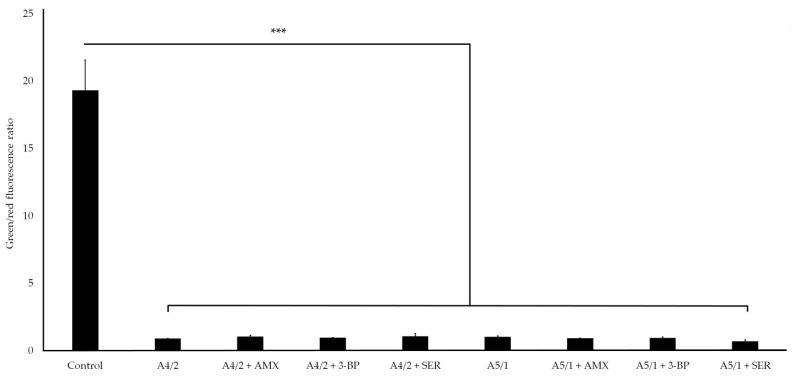
Viability of *H. pylori* 8064 biofilm cells treated with the tested plant extracts (A4/2 or A5/1) and their combination with tested synthetic compounds (amoxicillin (AMX), 3-bromopyruvate (3-BP), or sertraline (SER)) during the Bioflux-generated flow conditions. Bacteria were treated with substances as follows: MIC of a plant extract, ¼ × MIC of a plant extract + ¼ × MIC of AMX, ¼ × MIC of a plant extract + ¼ × MIC of 3-BP, or ¼ × MIC of a plant extract + ½ × MIC of SER. The study controls were microcapillaries colonized by bacteria not exposed to any antimicrobial substances. Viability was determined by measuring the green/red fluorescence ratio of bacteria stained with the LIVE/DEAD kit. The *p*-value represented by *** is equal to <0.0001.

In the first stage of the research measuring the antibiofilm activity of extracts and their combination with AMX, 3-BP, or SER, the activity of these components was verified using the Bioflux 1000 system. This modern, automatic system is able to set precisely a speed of medium flow; thanks to that, it allows to obtain more complex data than stationary experimental set-ups, such as commonly applied microtitration plate models [50,51]. In this context, it is also worth mentioning that this research is the first in which flow conditions were applied to determine the antibiofilm activity of substances against *H. pylori*. After a 24-h incubation of *H. pylori* 8064 with tested substances in the Bioflux system, we noticed a significant reduction in the biofilm level of the analyzed strain compared with the control setting in which biofilm was non-exposed to any challenges (*p*-value < 0.0001; Figure 2, Figure 3, Figure 4 and Figure 5). For bacteria treated with the MIC of A4/2, biofilm development was reduced to 75%, and much more strongly when ¼ × MIC of the extract was combined with one of the tested synthetic substances (¼ × MIC of AMX or 3-BP, or ½ × MIC of SER) (Figure 2 and Figure 4). In this case, the level of *H. pylori* biofilm constituted only 23–38% of an untreated setting. The strong antibiofilm activity was also found for the second of the tested extracts, A5/1, because both alone and in the combination with AMX, 3-BP, or SER, the level of biofilm decreased to 33–42.5% compared with the untreated setting (Figure 3 and Figure 4).

Additionally, we determined the fluorescence of biofilm cells stained with the LIVE/DEAD kit to assess the viability of this structure (Figure 5). For all samples exposed to the tested extracts/substances, a significant reduction in the green/red fluorescence ratio of treated vs. untreated biofilms was observed (0.69–1.06 vs. 19.3; *p*-value < 0.0001), indicating a decrease in the viability of bacterial cells and high activity of extracts and their combinations with AMX, 3-BP, and SER against planktonic cells and the initial stages of *H. pylori* biofilm development.

**Figure 6:**
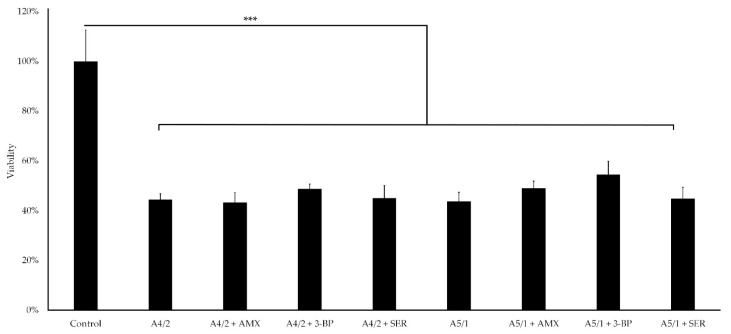
Viability of *H. pylori* 8064 lawn biofilms treated with cellulose disks chemisorbed with 1 mg of the tested plant extracts (A4/2 or A5/1) or an appropriate weight combination with tested synthetic compounds (amoxicillin (AMX), 3-bromopyruvate (3-BP), or sertraline (SER)) determined on the basis of checkerboard assays (0.25 mg of a plant extract + 0.25 mg of AMX, or 0.25 mg of a plant extract + 0.25 mg of 3-BP, or 0.25 mg of a plant extract + 0.5 mg of SER). Disks with 1% DMSO were the negative control of the studies. The *p*-value represented by *** is equal to <0.0001.

In the second step of determining the antibiofilm activity of the tested components, the antimicrobial effect of the extracts and their combinations with AMX, 3-BP, or SER was checked against a mature 3-day-old biofilm growing in the form of lawn. At this stage, we used a model established by our research group and presented in previous studies [22,52], involving the sorption of the tested components into cellulose disks and determining their antibiofilm activity against biofilm formed on porous, soft agar surface. The application of this in vitro model is particularly useful for testing substances that should be applied locally (they should not/cannot be used in systemic therapies, e.g., due to inability to achieve an appropriate bactericidal concentration) and require an appropriate delivery carrier [31,32]. Biocellulose appears to be a suitable type of antimicrobial drug carrier thanks to its numerous beneficial properties, including non-toxicity, high drugs absorption, and high mechanical/chemical resistance [53,54,55]. Prior to the main part of the research, the disk-diffusion method with cellulose disks chemisorbed alone with A4/2 or A5/1 extracts or their combinations with AMX, 3-BP, or SER was performed (Table S1 and Figure S3). The visible zones of growth inhibition in the range of 24 ± 1 mm to 75.3 ± 1.5 mm confirmed that the cellulose disks had the ability to absorb and release the compounds we tested. In the next stage of this research, we examined the antimicrobial activity of these disks against mature bacterial biofilm formed by *H. pylori* 8064 (Figure 6). The viability expressed as CFU/mL of *H. pylori* 8064 biofilm cells decreased to 43.2–54.5% compared with the control setting (*p*-value < 0.0001). This effect indicated the ability of the tested substances to penetrate the microbial biofilm barrier and eradicate a substantial proportion of bacterial cells. Remarkably, this effect was achieved after a single, 4-h exposure of *H. pylori* 8064 biofilm to cellulose disks loaded with the tested compounds. Hence, we presume that the repeated treatment of the biofilms for several days could significantly increase the observed antibacterial effect. The usefulness of the carriers, including biocellulose, shall be emphasized in terms of combating microbes and potentially reducing cytotoxicity to the host. These results and the utility of the above carrier-drug systems can be very effective in the treatment of *H. pylori* infections, although they need further validation in vivo to confirm their applicability.

### 2.5. Limitations and Future Perspectives

However, some limitations to the above presented results should be borne in mind before continuing to use the tested herbs and the extracts thereof as potential *H. pylori* eradicating agents, alone or with combination with synthetic drugs.

The study was performed using a single *H. pylori* strain that was dictated by the necessity to focus on several different aspects, such as (1) general antimicrobial action of the extracts against both planktonic forms and biofilm (present in two forms: aggregates or lawn), (2) synergistic activity of plant extracts with substances of known antimicrobial activity, (3) effect of extracts after their chemisorption on cellulose disks, and (4) cytotoxicity of extracts towards eukaryotic cells. All these aspects contribute to the understanding of the activity of the extracts against *H. pylori* but would be difficult to obtain in a single study if incorporating several strains of the same species. Nonetheless, the observed outcome needs verification using carefully selected, additional strains before drawing final clinically relevant conclusions.

Furthermore, a possibility of developing or acquiring resistance to the active constituents is a potential risk in successful implementation in anti-*H. pylori* therapy. In our study, a potential countermeasure was to use the complex extractives (consisting of several alkaloids) additionally combined with synthetic drugs. However, as this potentially undesired effect was not actually studied, it opens an opportunity for further investigation into the mechanisms by which the pathogenic bacteria may cope with such combined therapeutics. Despite many reports on differences between microbial strains in sensitivity to plant extracts, no data were found in the literature on specific mechanisms that would confer the resistance against complex mixtures of natural products containing both alkaloids and polyphenols [56]. Certainly, this issue needs to be addressed in future research aiming at verification of whether *H. pylori* is able to develop resistance to these and other plant-derived antibacterials and what would be the potential mechanisms of it.

Finally, the cytotoxicity against stomach- and liver-derived cells may be associated with an inflammatory response manifested by cytokine expression and release. In our previous study on lipopolysaccharide-stimulated human neutrophils [57], we found that several alkaloids (sanguinarine, berberine, chelidonine, and chelerythrine) contained in the extracts from both tested plants [56], depending on the specific concentrations, had a dual anti-inflammatory and pro-inflammatory effect. Hence, the inflammatory response of stomach and liver cells and tissues upon treatment with the antibacterial extracts should be considered in future studies, including in vivo models.

## 3. Materials and Methods

### 3.1. Plant Material

Six Papaveraceae species were selected for testing their antimicrobial properties. Plants were collected from four locations: (I) the Botanical Garden of Medicinal Plants in Wroclaw, geographical location: 51.117121,17.074088 (*Chelidonium majus*—C.m., *Corydalis cheilanthifolia*—C.ch., *Fumaria vaillantii*—F.v.); (II) the Botanical Garden of Maria Curie-Skłodowska University at Lublin, geographical location: 51.2657359,22.5144798 (*Pseudofumaria lutea*—P.l.); (III) Jagodno-Wroclaw, geographical location: 51.0553649,17.0576109 (*Fumaria officinalis*—F.o.); (IV) Botanical Garden of Jagiellonian University, Kraków, geographical location: 50.0635703,19.9533424 (*Glaucium flavum*—G.f.). Flowering plants were harvested in April 2018: *C.majus* on 27 April; *C.cheilanthifolia* and *F.vaillantii* on 21 April. Flowering *P. lutea* was harvested on 20 April 2018, as previously reported by Zielińska et al. (2020) [18] and flowering *F. officinalis* was harvested on 13 May 2020 and flowering *G. flavum* was harvested on 3 June 2020. Voucher specimens of the species were deposited in the Herbaria of Botanical Gardens, described above, under the codes: L02/C.m.1-15/18; L01/C.ch.1-11/18; L01/F.v.1-10/18; P.l.—4223A; L01/F.o.1-10/20; G.f.—OB-6777-R, respectively. For obtaining extracts, the plant material was dried at 25–35 °C, separated into aerial and underground parts and ground in mortar.

The 200 mg of dried plant material was extracted in ultrasound bath (IS-10R, Intersonic, Olsztyn, Poland) using 5 mL of solvent solution (MeOH acidified with 0.1% formic acid in the ratio of 4:1) in total. The extraction was performed twice, and the supernatants were combined every time after centrifugation. The extracts were dried under the sample concentrator with nitrogen (VLM GmbH, 33,689 Bielefeld Heideblumchenweg 50, Type: V.569.061.820, 230V, 600W, 50–60 Hz, Germany). The obtained dry extract yield was as follows: C.m. herb—141.6 mg, C.m. roots—86.7 mg; C.ch. herb—120.9 mg, C.ch. roots—103.1 mg; F.v. herb—101.8 mg, F.v. roots—95.8 mg; P.l. herb—99.5 mg, P.l. roots—70.2 mg; F.o. herb—146.1 mg, F.o. roots—62.2 mg; G.f herb—99.4 mg, G.f. roots—112.3 mg.

### 3.2. Determination of the Antimicrobial Activity against H. pylori

#### 3.2.1. Bacterial Strain

During all stages of the research, the multidrug-resistant, clinical *H. pylori* 8064 strain was used, for which resistance to CLR, LEV, and MTZ was proved in our earlier research [22]. This strain was kept in Tryptic Soy Broth (TSB; Oxoid, Dardilly, France) with 15% glycerol in a freezer at −70 °C [34,35]. The revival of the strain was accomplished by sowing frozen samples on Columbia agar (Difco, Lublin, Poland) enriched with 7% horse blood. The bacteria were grown for 3–5 days under microaerophilic conditions (Genbox microaer kits, BioMerieux, Marcy-l’Étoile, France) at 37 °C.

#### 3.2.2. Broth Microtitration and Spot Assays

In order to determine minimum inhibitory concentrations (MICs) and minimum bactericidal concentrations (MBCs), the microtitration and spot methods were used, respectively [34,35]. The research was carried out in ventilated 12-well titration plates (Bionovo, Legnica, Poland) with wells filled with 1 mL of Brain Heart Infusion broth (BHI; Oxoid, Dardilly, France) and 5% fetal calf serum (FCS; Gibco, Paisley, Scotland, UK), bacteria with a density of 10^7^ CFU/mL, and a concentration gradient of the tested plant extracts (4–512 µg/mL). These titration plates were then directed to a 3-day microaerophilic culture at 37 °C and 100 rpm (0.1× *g*) shaking (MaxQ 6000, ThermoFisher, Waltham, MA, USA). After this step, bacterial growth was verified in all wells, and the lowest concentration in which no turbidity of the medium was noticed was classified as the MIC. From each well, 10 µL of the suspension was then spotted on Columbia agar with 7% horse blood and incubated for a further 3 days under microaerophilic conditions at 37 °C. The spot with the lowest concentration of the extract without bacterial colonies growth was interpreted as the MBC.

#### 3.2.3. Checkerboard Assays

Determination of interactions between the selected plant extracts and substances with documented antimicrobial activity against *H. pylori* 8064 (amoxicillin (AMX), 3-bromopyruvate (3-BP), or sertraline (SER)) [22,34,35], all from Sigma-Aldrich (St. Louis, MO, USA) was performed using the checkerboard assay [34,35]. To obtain this, four 12-well plates were joined together to form a 48-well panel. Each well of the titration plates was filled with 1 mL of BHI with 5% fetal calf serum, bacteria with a density of 10^7^ CFU/mL, and a mixture of both tested components (one of the extracts (corresponding to MIC–1/64 × MIC) and one of the aforementioned synthetic substance (corresponding to MIC–1/16 × MIC)). Such titration plates were then directed to a 3-day microaerophilic culture at 37 °C and 100 rpm (0.1× *g*) shaking. The interactions between the tested components were determined by calculating the fractional inhibitory concentration index (FICI), for which the values ≤0.5, 0.5–1, and >1 were considered as synergistic, additive, and neutral, respectively [33].

#### 3.2.4. Paper and Biocellulose Disk-Diffusion Assays

The activity of selected plant extracts was additionally verified using the paper and cellulose disk-diffusion method [22]. The cellulose used for the production of the disks came from the stationary phase growth of *Komagataeibacter xylinus* DSM 46602, and its chemical purification was made according to the procedure established by Junka et al. (2017) [58]. The tested substances were dissolved in dimethyl sulfoxide (DMSO; Sigma-Aldrich, St. Louis, MO, USA) in such a way that the final concentration of this solvent did not exceed 1% and did not interfere with the bacterial growth. A single paper (6 mm) or cellulose (15 mm) disk was chemisorbed with 1 mg of the extract/substance or an appropriate weight combination determined on the basis of checkerboard assays (0.25 mg of a plant extract + 0.25 mg of AMX, or 0.25 mg of a plant extract + 0.25 mg of 3-BP, or 0.25 mg of a plant extract + 0.5 mg of SER) and placed in the center of a plate with Columbia agar and 7% horse blood containing a seeded lawn of bacteria. The negative control of the tests were disks with 1% DMSO (both paper and cellulose) and ready-made paper disks with CLR, MTZ, or LEV (antibiotics against which the *H. pylori* 8064 strain is resistant) (Oxoid, Dardilly, France). All culture plates were incubated under standard conditions (microaerophilic atmosphere at 37 °C) for 3 days.

#### 3.2.5. Antibiofilm Assays

The activity of selected plant extracts with or without synthetic substances (AMX, 3-BP, or SER) was determined using two different methods, i.e., the analysis of microcapillary overgrowth under flow conditions, determining the effect of the tested compounds on the first stages of biofilm formation, as well as the modified lawn biofilm assay to determine the effect of the tested compounds on the mature biofilm.

The development of the first stages of biofilm and the degree of microcapillaries coverage was determined using the Bioflux 1000 automatic system (Fluxion, San Francisco, CA, USA). The dedicated 48-well microfluidic plates (Fluxion, San Francisco, CA, USA), compatible with this system, consist of two wells (inlet and outlet) and a microcapillary connecting both wells. At the beginning, 0.9 mL of BHI broth with 5% fetal calf serum and the appropriate concentration of the tested plant extracts with or without synthetic substances was added to the inlet wells (i.e., MIC of a plant extract, ¼ × MIC of a plant extract + ¼ × MIC of AMX, ¼ × MIC of a plant extract + ¼ × MIC of 3-BP, or ¼ × MIC of a plant extract + ½ × MIC of SER). The study controls were microcapillaries colonized by bacteria not exposed to any antimicrobial substances. Then, the aforementioned medium was passed through a microcapillary (inlet to outlet) at 10 dyne/cm^2^ (1060 µL/h) for 10 s, followed by a 15 min incubation to allow the medium components to pre-coat the microcapillary surface. In the next step, 0.1 mL of bacterial suspension containing 10^8^ CFU/mL was added to the inlet wells, thereby obtaining 10^7^ CFU/mL in the well. The medium flow from the inlet to the outlet was turned on with the intensity of 0.1 dyne/cm^2^ (10.6 µL/h) for 24 h at 37 °C and microaerophilic atmosphere (Pecon Incubator XL S1, Carl Zeiss, Jena, Germany). After one day of incubation under these conditions, the medium flow was stopped and the inlet well was emptied from the remaining medium and then filled with 0.1 mL of a saline solution with 0.6 µL of 1:1 LIVE/DEAD dye mixture (Thermo Fisher, Waltham, MA, USA). The flow was turned back on in the inlet to outlet direction for 1 h to stain bacterial biofilms. After this step, pictures were taken with an inverted Carl Zeiss Microscopy (GmbH, Jena, Germany). The percentage of the microcapillary cover as well as the ratio of green to red fluorescence (interpreted as the ratio of live to dead bacteria) was calculated using the Bioflux Montage software (Fluxion, San Francisco, CA, USA).

The methodology of Krasowski and Junka et al. (2019) [52] and Krzyżek and Junka et al. (2020) [22], with minor modifications, was used to assess the activity of substances in relation to the mature biofilms. The 12-well titration plates were filled with 2 mL of BHI agar (Oxoid, Dardilly, France) and the agar was allowed to solidify, after which 15 mm diameter wells were cut in everywhere. In each of these wells, a 10 mm^2^ fragment of Columbia agar with 7% horse blood lawned by a 3-day *H. pylori* growth was placed in the hollow space. The hollow space containing the agar fragment with *H. pylori* lawn was then filled with BHI broth with 5% fetal calf serum to obtain a raised meniscus (approx. 0.5 mL). In the final stage, the place of the hollow was covered with a cellulose disk chemisorbed with 1 mg of the extract/substance or an appropriate weight combination determined on the basis of checkerboard assays (0.25 mg of a plant extract + 0.25 mg of AMX, or 0.25 mg of a plant extract + 0.25 mg of 3-BP, or 0.25 mg of a plant extract + 0.5 mg of SER). Disks with 1% DMSO were the negative control of the studies. The plates prepared in such manner were then incubated for 4 h at 37 °C under microaerophilic conditions and 50 rpm (0.027× *g*) shaking. After this time, the bacterial viability was determined by homogenizing the agar pieces with bacterial lawn in 1 mL of BHI with 5% fetal calf serum. Next, a series of dilutions were made, and 0.1 mL of the suspension was plated on Columbia agar plates with 7% horse blood. The plates were incubated for 7 days under standard conditions (microaerophilic atmosphere at 37 °C).

### 3.3. Cytotoxicity In Vitro against Human Cell Lines

The normative neutral red cytotoxicity assay was performed for hepatic (HepG2; ATCC HB-8065, Manassas, VA, USA) and gastric (AGS; ATCC CRL-1739, Manassas, VA, USA) cell cultures treated with tested plant extracts (extracts from the underground parts of *C. majus* (A4/2) and the aerial parts of *C. cheilanthifolia* (A5/1)) [59,60]. Cells were seeded onto a 96-well microtitration plate at density of 2 × 10^4^ cells per well in a high glucose DMEM culture medium (Dulbecco’s modified Eagle’s medium; Gibco, Paisley, Scotland, UK) or F-12 medium (Biowest LLC, Riverside, MO, USA) supplemented with 10% bovine serum (Gibco, Paisley, Scotland, UK) and antibiotics (penicillin 100 U/mL and streptomycin 0.1 mg/mL, both from Gibco, Paisley, Scotland, UK). After 72 h of microaerophilic culture at 37 °C, the cells were stimulated with 4-fold serial dilutions of the analyzed extracts for an additional 24 h. Cells treated with 70% ethanol (Stanlab, Lublin, Poland) for 3 min were the negative control. Next, the examined solutions were removed and neutral red (40 µg/mL in PBS; Sigma-Aldrich, St. Louis, MO, USA) was added to the wells in a volume of 0.1 mL and incubated for 2 h at 37 °C and microaerophilic conditions. After this step, the dye solution was removed and cells were washed with PBS and dried. Subsequently, 0.15 mL of a solution containing 70% ethanol, deionized water, and glacial acetic acid mixed in a 50:49:1 (*v/v*) (POCH, Gliwice, Poland) was added to each well to extract neutral red. The extracts in appropriate dilutions, introduced to a 96-well microtitration plate, served as blank measurements. After vigorous plate shaking for 30 min, neutral red absorbance was measured using a Synergy H4 microplate reader (BioTek, Winooski, USA) at OD_540_.

### 3.4. Statistical Analysis

Calculations were performed using the GraphPad Prism version 7 software (GraphPad Co., San Diego, CA, USA). The normality of distribution was assessed by means of the D’Agostino–Pearson omnibus test. Because all values were non-normally distributed, the Kruskal–Wallis test with the post hoc Dunnett analysis was applied. The results of statistical analyses were considered significant if they produced *p*-values < 0.05.

## 4. Conclusions

The results of the current in vitro study showed a potent antimicrobial activity of plant extracts from *C. majus* and *C. cheilanthifolia* alone and in combination with tested synthetic substances (amoxicillin, 3-bromopyruvate, or sertraline) against planktonic and biofilm forms of *H. pylori*. Combining the techniques of using complex matrices, such as bio-carriers chemisorbed with mixtures of plant natural products and synthetic compounds, gives an opportunity for multidirectional influence on pathogen cells and may be considered as a new, promising solution for the eradication of biofilm-forming *H. pylori*.

## Figures and Tables

**Table 1:** Antibacterial activity of tested extracts against *H. pylori* 8064.

Plant Species	Part of the Plant	Designation	Antibacterial Activity (µg/mL)	
			MIC	MBC
*Glaucium flavum*	Aerial	A1/1	512	>512
	Underground	A1/2	512	>512
*Fumaria officinalis*	Aerial	A2/1	>512	>512
	Underground	A2/2	>512	>512
*Fumaria vailantii*	Aerial	A3/1	>512	>512
	Underground	A3/2	>512	>512
*Chelidonium majus*	Aerial	A4/1	512	>512
	Underground	A4/2	**128**	**256**
*Corydalis cheilanthifolia*	Aerial	A5/1	**64**	**64**
	Underground	A5/2	**64**	**128**
*Pseudofumaria lutea*	Aerial	A6/1	>512	>512
	Underground	A6/2	>512	>512

Abbreviations: MIC, minimal inhibitory concentration; MBC, minimal bactericidal concentration.

**Table 2:** Zones of growth inhibition caused by the tested extracts and clinically used antibiotics against *H. pylori* 8064.

Antimicrobial Compound	Dose	Growth Inhibition Zone (mm)
Tested plant extracts		
A4/2	1 mg	22.7 ± 2.5
A5/1	1 mg	30.7 ± 0.6
A5/2	1 mg	28 ± 1
Antibiotics		
CLR	15 µg	11.3 ± 1.5
LEV	5 µg	6
MTZ	5 µg	6
AMX (a positive control)	25 µg	55.7 ± 2.1
Empty disk (a negative control)	-	6

The abbreviations of antibiotic disks used: CLR, clarithromycin; LEV, levofloxacin; MTZ, metronidazole; AMX, amoxicillin.

**Table 3:** Interactions determined by the checkerboard method between selected plant extracts (A4/2 and A5/1) and selected synthetic substances (amoxicillin, 3-bromopyruvate, and sertraline) against *H. pylori* 8064.

Tested Combination	MIC (µg/mL)						FICI (Outcome)
	Selected Plant Extract			Selected Synthetic Compound			
	Alone	Combination	Fold Change	Alone	Combination	Fold Change	
A4/2 + AMX	128	32	4	0.06	0.015	4	0.5 (synergistic)
A4/2 + 3-BP	128	32	4	128	32	4	0.5 (synergistic)
A4/2 + SER	128	32	4	2	1	2	0.75 (additive)
A5/1 + AMX	64	16	4	0.06	0.015	4	0.5 (synergistic)
A5/1 + 3-BP	64	16	4	128	32	4	0.5 (synergistic)
A5/1 + SER	64	16	4	2	1	2	0.75 (additive)

Abbreviations: AMX, amoxicillin; 3-BP, 3-bromopyruvate; SER, sertraline; MIC, minimal inhibitory concentration; FICI, fractional inhibitory concentration index.

## Data Availability

Not applicable.

## Supplementary Materials

The following are available online at https://www.mdpi.com/article/10.3390/pathogens10081033/s1, Figure S1: Representative photos of plates containing *H. pylori* 8064 lawn and disks saturated with tested plant extracts or antibiotics, Figure S2: Viability of hepatic and gastric cell cultures treated with tested plant extracts measured by the normative neutral red cytotoxicity assay, Figure S3: Representative photos of plates containing *H. pylori* 8064 lawn and cellulose disks chemisorbed with tested plant extracts and their combination with selected synthetic compounds, Table S1: Zones of growth inhibition caused by cellulose disks chemisorbed with selected plant extracts of the Papaveraceae family and selected synthetic substances against *H. pylori* 8064.
