# Supplementary material for: Antibiofilm and Antimicrobial-Enhancing Activity of Chelidonium majus and Corydalis cheilanthifolia Extracts against Multidrug-Resistant Helicobacter pylori
Source: Pathogens. 2021 Aug 16;10(8):1033. doi: 10.3390/pathogens10081033 (PMC8400265; doi:10.3390/pathogens10081033)
Supplement: Supplementary file 1 [file pathogens-10-01033-s001.zip › pathogens-1334567-SI.pdf]

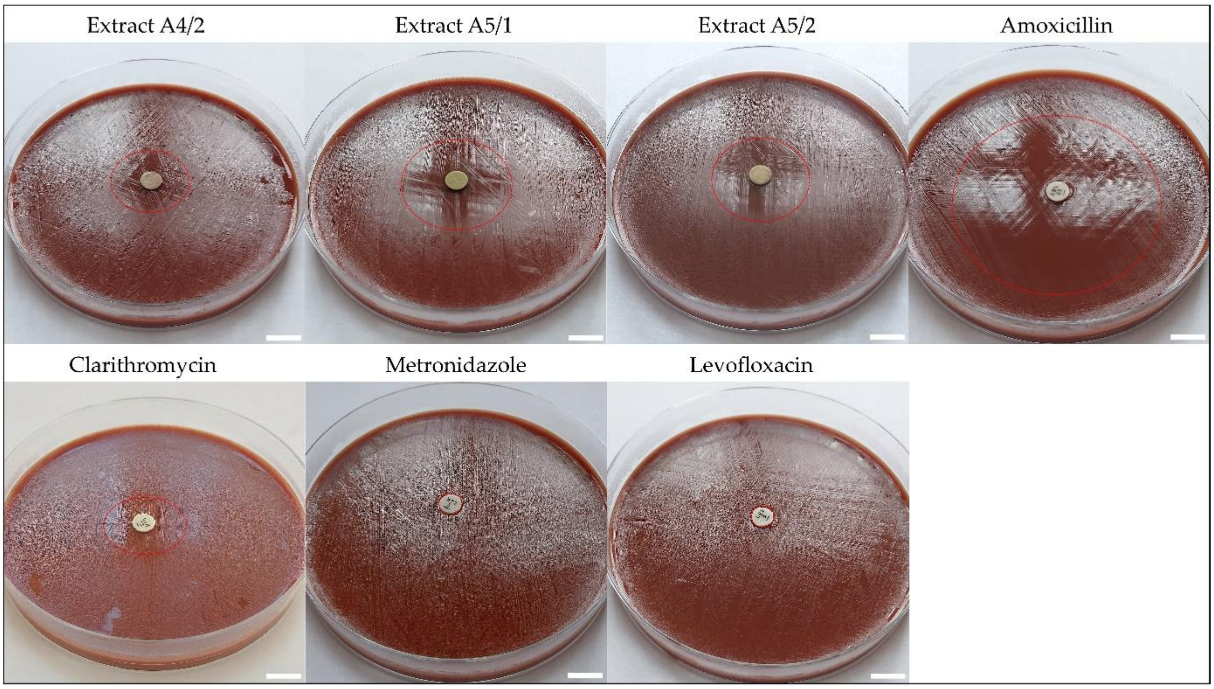

Figure S1. Representative photos of plates containing *H. pylori* 8064 lawn and disks saturated with tested plant extracts (A4/1, A5/1, and A5/2) or antibiotics (amoxicillin, clarithromycin, metronidazole, and levofloxacin). The antimicrobial activity was determined by measuring the size of growth inhibition zones. Amoxicillin was the positive control, while clarithromycin, metronidazole and levofloxacin served as negative controls. Scale bars are equal to 1 cm.

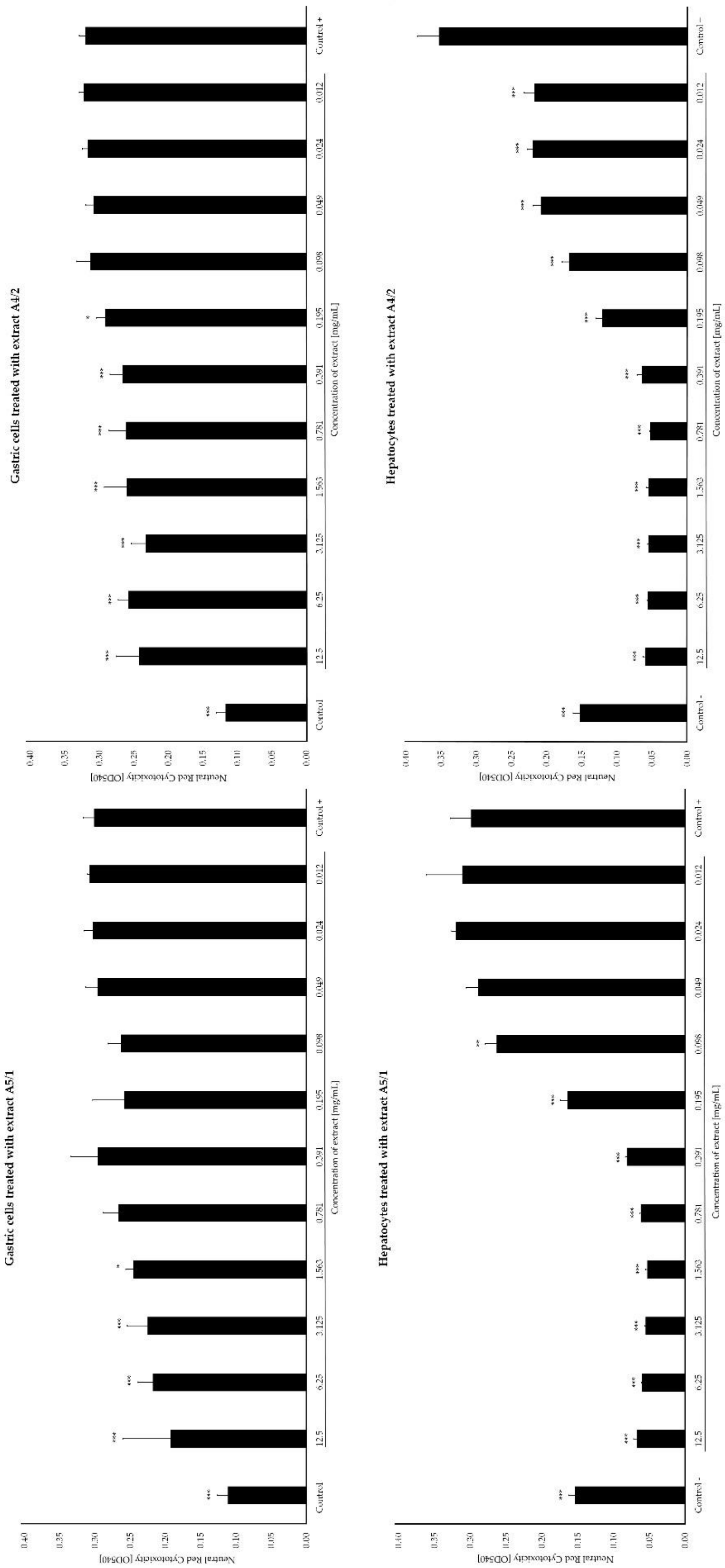

Figure S2. Viability of hepatic (HepG2; ATCC HB-8065) and gastric (AGS; ATCC CRL-1739) cell cultures treated with tested plant extracts (extracts from the underground parts of *C. majus* [A4/2] and the aerial parts of *C. cheilanthifolia* [A5/1]) measured by the normative neutral red cytotoxicity assay. Cells treated with 70% ethanol and not treated with any extract were negative and positive controls, respectively. The *p*-values represented by ***, ** and * were equal to < 0.0001, < 0.001 and < 0.05, respectively.

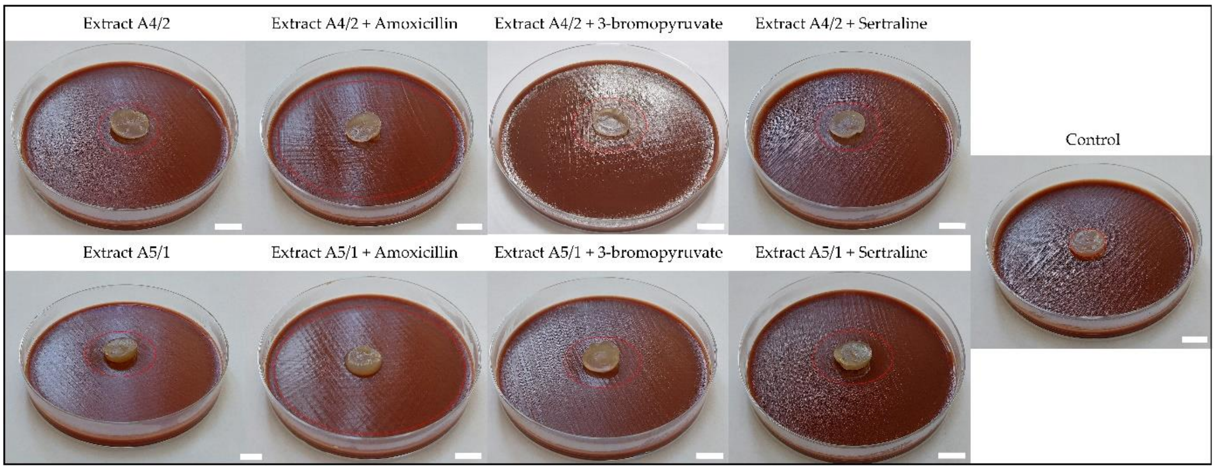

Figure S3. Representative photos of plates containing *H. pylori* 8064 lawn and cellulose disks chemisorbed with 1 mg of tested plant extracts (A4/1 and A5/1) and their combination with selected synthetic compounds (amoxicillin (AMX), 3-bromopyruvate (3-BP), or sertraline (SER)) determined on the basis of checkerboard assays (0.25 mg of a plant extract + 0.25 mg of AMX, or 0.25 mg of a plant extract + 0.25 mg of 3-BP, or 0.25 mg of a plant extract + 0.5 mg of SER). Disks with 1% DMSO were the negative control of the studies. The antimicrobial activity was determined by measuring the size of growth inhibition zones. Scale bars are equal to 1 cm.

Table S1. Zones of growth inhibition caused by cellulose disks chemisorbed with selected plant extracts of the Papaveraceae family (A4/2 and A5/1) and selected synthetic substances (amoxicillin, 3-bromopyruvate and sertraline) against *H. pylori* 8064.

Antimicrobial compound	Dose *	Growth inhibition zone [mm]
A4/2	1 mg	24 ± 1
A4/2 + AMX	0.25 mg + 0.25 mg	70.7 ± 2.5
A4/2 + 3-BP	0.25 mg + 0.25 mg	27.7 ± 1.5
A4/2 + SER	0.25 mg + 0.5 mg	28.3 ± 2.1
A5/1	1 mg	33.7 ± 1.5
A5/1 + AMX	0.25 mg + 0.25 mg	75.3 ± 1.5
A5/1 + 3-BP	0.25 mg + 0.25 mg	29 ± 1.7
A5/1 + SER	0.25 mg + 0.5 mg	28.3 ± 2.5
Empty disk (a negative control)	-	15

* Weight combinations were determined on the basis of results from checkerboard assays. Abbreviations: AMX, amoxicillin; 3-BP, 3-bromopyruvate; SER, sertraline.
